# Establishing New Mappings between Familiar Phones: Neural and Behavioral Evidence for Early Automatic Processing of Nonnative Contrasts

**DOI:** 10.3389/fpsyg.2016.00995

**Published:** 2016-06-30

**Authors:** Shannon L. Barrios, Anna M. Namyst, Ellen F. Lau, Naomi H. Feldman, William J. Idsardi

**Affiliations:** ^1^Department of Linguistics, University of UtahSalt Lake City, Utah, USA; ^2^Department of Linguistics, University of Maryland, College ParkCollege Park, MD, USA; ^3^Institute for Advanced Computer Studies, University of Maryland, College ParkCollege Park, MD, USA

**Keywords:** L1 Spanish, L2 English, L1 allophones, novel contrasts, MMN, allophonic split, perceptual categorization, phonological status

## Abstract

To attain native-like competence, second language (L2) learners must establish mappings between familiar speech sounds and new phoneme categories. For example, Spanish learners of English must learn that [d] and [ð], which are allophones of the same phoneme in Spanish, can distinguish meaning in English (i.e., /deɪ/ “day” and /ðeɪ/ “they”). Because adult listeners are less sensitive to allophonic than phonemic contrasts in their native language (L1), novel target language contrasts between L1 allophones may pose special difficulty for L2 learners. We investigate whether advanced Spanish late-learners of English overcome native language mappings to establish new phonological relations between familiar phones. We report behavioral and magnetoencepholographic (MEG) evidence from two experiments that measured the sensitivity and pre-attentive processing of three listener groups (L1 English, L1 Spanish, and advanced Spanish late-learners of English) to differences between three nonword stimulus pairs ([idi]-[iði], [idi]-[iɾi], and [iði]-[iɾi]) which differ in phones that play a different functional role in Spanish and English. Spanish and English listeners demonstrated greater sensitivity (larger d' scores) for nonword pairs distinguished by phonemic than by allophonic contrasts, mirroring previous findings. Spanish late-learners demonstrated sensitivity (large d' scores and MMN responses) to all three contrasts, suggesting that these L2 learners may have established a novel [d]-[ð] contrast despite the phonological relatedness of these sounds in the L1. Our results suggest that phonological relatedness influences perceived similarity, as evidenced by the results of the native speaker groups, but may not cause persistent difficulty for advanced L2 learners. Instead, L2 learners are able to use cues that are present in their input to establish new mappings between familiar phones.

## Introduction

Linguistic experience shapes listeners' sensitivities to phonetic distinctions. Specifically, extensive experience with one's native language (coupled with a lack of experience with nonnative sounds and contrasts) limits listeners' sensitivity to nonnative phonemic distinctions (Lisker and Abramson, 1970; Goto, 1971; Werker et al., 1981; Näätänen et al., 1997, to name just a few). This differential sensitivity to native vs. nonnative speech contrasts develops very early in life (Werker and Tees, 1984; Kuhl et al., 1992; Polka and Werker, 1994), and shapes the initial stages of second language (L2) speech perception (Escudero, 2005; Best and Tyler, 2007). These findings, and many others like them, have led to the development of models of cross-language and L2 speech perception and production (Best, 1995; Flege, 1995; Iverson et al., 2003; Escudero, 2005; Best and Tyler, 2007) which make predictions about how naive nonnative and L2 listeners will perceive and acquire target language sounds and contrasts. More recently, however, there has been growing interest in how allophones (i.e., phones which are present in the ambient language, but which are not used to distinguish word meanings) are represented and processed by adults (Kazanina et al., 2006; Boomershine et al., 2008; Johnson and Babel, 2010), and how this knowledge of phonological status develops in infants (Seidl and Cristia, 2012).

The present study contributes to this literature on sound category learning by investigating the role of language-specific phonological patterning in L2 phonological development. We use both behavioral methods and magnetoencepholographic (MEG) recordings to investigate how adult second language learners' knowledge of native language (L1) phonological patterns impacts the acquisition of their second language sound system. In particular, we ask whether advanced adult late-learners of a second language overcome native language mappings to establish new phonological relations between familiar phones.

**Figure 1:**
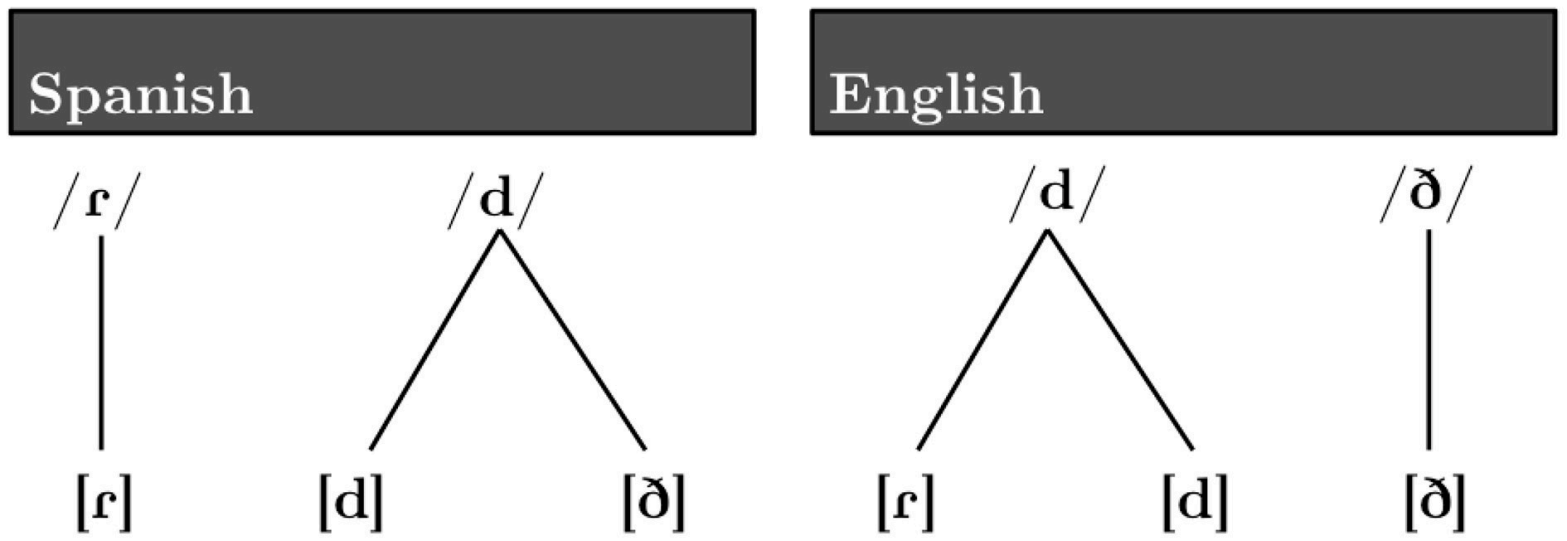
**Relation between allophones and phonemes in Spanish (***left***) and English (***right***)**.

Languages differ in their mappings between predictable surface variants (i.e., allophones) and more abstract phonological categories (i.e., phonemes) (Kenstowicz, 1994). Consider, for example, the relation between the phonological systems of Spanish and English (Figure 1), in which sets of sound categories with very similar acoustic distributions map onto different sets of phonemes in the two languages.

Although three very similar phonetic categories, [d], [ð], and [ɾ], exist in both Spanish and English, the functional significance of these categories varies between the two languages. The phones [d] and [ð] distinguish word meanings in English (i.e., [ðeɪ] “they” and [deɪ] “day”). In contrast, a productive phonological pattern causes the voiced obstruents /b, d, ɡ/ to surface as the approximants [β, ð, ɣ] intervocalically in Spanish^1^. Thus, whereas [d] and [ð] are contrastive in English, the two distinct acoustic realizations are phonologically conditioned variants (allophones or positional variants) of the same phoneme category in Spanish. An important component of native speakers' knowledge of an allophonic alternation of this sort is that allophonic variants are tied to particular phonological contexts, whereas the phoneme is not. Thus, native Spanish speakers have internalized knowledge of the contexts in which the allophonic variants [d] and [ð] occur. While the exact pattern of allophony is known to vary by Spanish dialect (See Carrasco et al., 2012 for a review of this literature, as well as acoustic analyses characterizing the differences between Costa Rican and Madrid varieties of Spanish), the approximant (rather than the stop) is expected intervocalically in all dialects. On the other hand, the phones [d] and [ɾ] are contrastive in Spanish, but not in English. In American English /d/ (and /t/) surface as [ɾ] in post-tonic intervocalic position, and [d] elsewhere (i.e., [ɹaɪ:d] “ride” vs. [ˈɹaɪ:ɾɚ] “rider”)^2^.

A consequence of cross-linguistic variation in the mapping between speech sounds and phonemes is that L2 learners may need to establish new mappings between familiar phones. For example, to attain native-like competence in English, a Spanish learner must learn that [d] and [ð], which are allophones of a single phoneme (i.e., /d/) in Spanish, can distinguish word meaning in English. Doing so is assumed to entail the updating of internalized knowledge about the distribution of the phones in the L2 (i.e., learning that the phones are not restricted to particular environments in the target language, but instead can occur in the same phonological environments). Eckman et al. (2001; 2003, and subsequent work) referred to this learning scenario, in which sounds that are allophones of one phoneme in a learner's native language constitute separate phonemes in the target language, as an ‘allophonic split’^3^. It is this L2 learning scenario that is the focus of the present study^4^.

L1 context-dependent allophones present unique challenges for the L2 learner from the perspective of production and perception. The learner must learn to detect the target language phonemic contrasts in perception, and suppress L1 positional variants in L2 production (even when the phonological context is appropriate for their production). Both anecdotal and experimental evidence from speech production (Lado, 1957; Hammerly, 1982; Hardy, 1993; Zampini, 1996; Eckman et al., 2001, 2003) suggests that this learning situation presents considerable difficulty for second language learners. However, the acquisition of novel target language contrasts between L1 context-dependent allophones has not been adequately explored from the perspective of L2 speech perception.

Research with adult native listeners has revealed that speech perception is not only influenced by listeners' experience (or lack of experience) with the phones in question; the phonological status of a sound contrast also affects listeners' perception. Several behavioral studies have reported differences in the perception of familiar phones (i.e., phones that occur regularly in the native language of the listener) depending on whether the sounds in the pair function as contrastive phonemes or non-contrastive allophones in the listener's native language. In particular, these studies report that sounds which are contrastive are discriminated more readily, and are rated less perceptually similar than allophonically related phones (Pegg and Werker, 1997; Whalen et al., 1997; Harnsberger, 2001; Peperkamp et al., 2003; Boomershine et al., 2008). For example, Whalen et al. (1997) used a categorical AXB task to investigate the discriminability of the phones [p], [p^h^], and [b] by adult English listeners. They found that English listeners more readily discriminated the distinction between phonemic contrasts [b]-[p] and [b]-[p^h^] than the allophonic contrasts [p]-[p^h^] in a word-medial syllable initial position.

A similar pattern was also reported by Pegg and Werker (1997), who used an AX discrimination task to compare listeners' sensitivity to the voiced and the voiceless unaspirated alveolar stop pair, [d]-[t], relative to the [d]-[t^h^] pair. Crucially, while adult English listeners have extensive experience with all three phones, [d]-[t^h^] serve to distinguish words in word-initial position in English, whereas [d]-[t] do not. In line with Whalen et al. (1997), the phonemic pair was discriminated more accurately than the allophonic pair, despite the listeners' extensive experience with both phones in perception and production.

Peperkamp et al. (2003) used an AX discrimination task to investigate French listeners' perception of phonemic [m]-[n] and allophonic [ʁ]-[χ] contrasts. In French, [χ] is a predictable variant of the phoneme /ʁ/ which precedes a voiceless consonant. Like the other studies mentioned above, the authors found better discrimination for the phonemic [m]-[n] pair than the allophonic distinction between [ʁ] and [χ], when the latter were presented in a preconsonantal environment (i.e., [aʁ.CV]-[aχ.CV]). Interestingly, poorer discrimination was observed for the allophonic contrast regardless of whether the voicing of the consonant in the context syllable was phonotactically legal (matched the phone in question in voicing) or not, suggesting that allophonic variants are represented as a single phonological category.

In a recent study, Boomershine et al. (2008) used a similarity rating and a speeded AX discrimination task to investigate the impact of contrast and allophony on the perception of the phones [d], [ð], and [ɾ] in intervocalic contexts by native English and Spanish listeners. The authors hypothesized that, if the phonological status of these segments in the listeners' native language determines the perceived similarity of the pair, we should expect relatively more discrimination difficulty (longer RTs on a speeded AX discrimination task) and greater perceived similarity (higher similarity ratings on a similarity rating task) for the allophonic than for phonemic contrasts. These predictions were borne out. Spanish listeners produced higher similarity ratings and longer RTs than English speakers for the [d]-[ð] contrast, which are allophones of the same phoneme in Spanish. In contrast, English listeners had more difficulty discriminating [d]-[ɾ], which are phonologically related in their native language. The pair was also rated by English listeners as being perceptually less distinct than the other two contrasts^5^. These findings are consistent with those reported earlier and provide additional evidence that listeners' perception is shaped by the phonology of their native language. In particular, the phonological status of pairs of phones in a listener's native language is an important factor in determining the discriminability and perceived similarity of a pair of phones (see also Johnson and Babel, 2010 who report data for Dutch listeners' perception of fricatives, Shea and Renauld, 2014 for Spanish listeners' perception of the palatal obstruent alternation, and Harnsberger, 2001 for Malayalam listeners' perception of allophonically-related dental and alveolar nasal consonants).

In addition to the behavioral studies reviewed above, research using neurophysiological techniques has also reported important differences in the processing of contrastive vs. non-contrastive sound pairs (Näätänen et al., 1997; Kazanina et al., 2006). Unlike behavioral measures, which may reflect late conscious processes, time-sensitive measures such as electroencephalography (EEG) and magnetoencepholography (MEG) measure neuronal activity in the brain directly and can be collected continuously without the necessity of an overt behavioral response on the part of the participant. They have thus proven useful for studying language processing and acquisition in a wide range of participant populations, including infants, and clinical populations. They also hold promise for studying language learners, since they may provide a measure of stimulus processing even in the absence of a behavioral change. For example, McLaughlin et al. (2004) demonstrated that ERPs to L2 words and pseudowords provide early evidence for word learning before changes in overt judgments were evident on lexical decision tasks. Therefore, it is possible that the learner's neural response will provide evidence of sound category learning that is not yet evident in her behavioral response.

A negative component of the event-related potential known as the mismatch negativity (MMN), and its magnetic counterpart, the mismatch field (MMF) response recorded using MEG, provide an early automatic, change detection response (Näätänen, 1992) which has proven useful for the study of auditory processing. The MMN is typically elicited in an oddball paradigm in which a stream of frequent repeated auditory stimulus (i.e., the standard in an experimental block) is interrupted by an oddball (i.e., an infrequent deviant acoustic event) which may differ in frequency, duration, intensity, phoneme category, etc. The MMN, which is obtained by subtracting the event-related response to the standard event from the response to the deviant event, typically peaks at 150–250 ms from the onset of an infrequent detectable change and can be elicited in the absence of attention (i.e., in passive listening conditions). Moreover, by making use of a paradigm in which participants are presented with multiple non-orthogonally varying tokens from each category (as opposed to an acoustic standard), an MMN serves as a measure of category identification (Phillips et al., 2000).

A number of studies have demonstrated that aspects of a listener's native phonology modulate MMN amplitude (Näätänen et al., 1997; Phillips et al., 2000; Kazanina et al., 2006). In a seminal study, Näätänen et al. (1997) investigated the role of experience with language-specific vowel categories by studying the MMN responses of Finnish and Estonian listeners to the Estonian vowels /e, ¨ o, ~ o, o/. Crucially, the Finnish language has the vowels /e, ¨ o, o/, but lacks /~o/. Finnish and Estonian listeners were presented with the vowel /e/ as the frequent standard stimulus and /¨o, ~ o, o/ as deviants in an oddball paradigm. The authors reported larger MMN responses for vowel contrasts involving native language vowel prototypes than contrasts involving nonnative vowel prototypes. That is, the Finnish participants showed an enhanced MMN response when the deviant vowel existed in Finnish, but the response was unexpectedly small (given the size of the acoustic difference in the F2 dimension) when it was elicited by a vowel that doesn't exist in the Finnish vowel inventory (i.e., /~o/), suggesting that the MMN response is influenced by experience with native language phoneme categories.

The MMN response has also been used as an index of nonnative vowel phoneme acquisition by second language listeners. Winkler et al. (1999) investigated whether novel vowel phoneme representations can be learned by recording the MMN responses of three groups of listeners, Finnish native speakers, proficient L1 Hungarian-L2 Finnish listeners, and naive L1 Hungarian listeners. The MMN responses of these groups were compared for two vowel contrasts, one that is phonemic in Finnish only (i.e., /e/-/æ/), and one that is phonemic in both languages (i.e., /e/-/y/). While an MMN was observed for all three groups for the /y/ deviants when presented in the context of the /e/ standard, the responses to /æ/ deviants differed as a function of experience. An MMN was observed for the /æ/ deviants for the native Finnish and the L1 Hungarian-L2 Finnish listeners, but not for the naive Hungarian listeners. This finding is taken to suggest that the proficient Hungarians had developed a new phonemic vowel representation for the Finnish vowel /æ/ as a result of their experience.

In a study which looked specifically at the pre-attentive processing of phonemes vs. allophones, Kazanina et al. (2006) investigated whether the MMF response is sensitive to the functional significance of native language sound categories. The authors examined the processing of the phones [t] and [d] in word initial position by Russian listeners, for whom the contrast is phonemic, and by Korean listeners, for whom the contrast is allophonic. That is, while both [t] and [d] naturally occur in word-initial position in Russian, only [t] is found word-initially in Korean. The voiced variant [d] occurs in intervocalic position in Korean. Thus, [t] and [d] do not distinguish meaning in Korean. Russian participants showed both behavioral evidence of categorical perception (i.e., a classic step-like identification function for the /ta/-/da/ VOT continuum and better between-category than within-category discrimination) and neurophysiological evidence of change detection in auditory cortex. In contrast, Korean participants showed neither behavioral, nor neurophysiological evidence of perceptual sensitivity to the pair. These results suggest that adult native listeners' auditory cortex groups sounds based on phonemic categories, and that the functional significance of sounds factors into speech perception at a very early stage of processing. Moreover, the amplitude of the MMN response can be used as an early automatic index of perceptual categorization.

In a recent training study with L2 learners, Herd (2011) made ERP recordings both prior to and following perception training in order to investigate the effects of training on the L1 English-L2 Spanish listeners' automatic, pre-attentive processing of auditory stimuli containing the Spanish /d/-/ɾ/ contrast. The author examined the processing of the phones [d] and [ɾ] in an intervocalic context (i.e., [ede] and [eɾe]) by Spanish listeners, for whom the contrast is phonemic, and by L1 English learners of Spanish, for whom the target language contrast is allophonic in their L1. As expected, native Spanish listeners showed a significant MMN response, with deviant stimuli eliciting a more negative response than their standard counterparts. This pattern was observed both when [ede] standard was compared to [ede] deviant and when [eɾe] standard was compared to [eɾe] deviant. L1 English learners of Spanish also showed a significant MMN for both pairs at post-test. Unexpectedly, however, an MMN response was also present at pre-test for [ede] standard vs. [ede] deviant for the L1 English learner group, suggesting that an [ede] deviant is detected in a stream of [eɾe] standards even before perception training. These results are difficult to interpret, however, since the author does not report the performance of a monolingual English control group. As a result, it is unclear how much learning has occurred, either prior to the training, or as a result of the training. More work is needed to understand the role of L1 context-dependent allophones in second language speech perception and phonological development.

A related question in bilingual speech perception has been whether early stages of speech representation which are indexed by the MMN can be affected by the language being used. For instance, in a follow up to their earlier study, Winkler et al. (2003) investigated whether Hungarian-Finnish bilinguals would show different patterns of neural activity in response to the same stimulus pairs as a function of language context. The authors elicited MMN responses with two oddball sequences in which the Finnish word /pæti/ “was qualified” served as the frequent standard stimulus and /peti/ “bed” the infrequent deviant, first in a Hungarian language context, and later in a Finnish language context. The Hungarian-Finnish bilingual participants exhibited an MMN response to the /pæti/-/peti/ pairs in both the Hungarian and Finnish contexts, and the responses elicited in the two contexts did not differ from one another. Based on these findings, the authors concluded that language context does not affect the automatic change detection response elicited by auditory deviance. Instead, the acquisition of a second language results in new phonemic categories that are used regardless of language context.

In contrast, a recent study by García-Sierra et al. (2012) demonstrated that language context can influence the pre-attentive detection of auditory deviance. The authors investigated Spanish-English bilinguals' MMN responses to two different pairings of three stimulus tokens from a synthetic VOT continuum in both a Spanish and an English language context. The language context was manipulated by having Spanish-English bilingual participants silently read magazines in either Spanish or English while ERPs were recorded. In the *phonemic in English* condition participants heard a stimulus token with +50 ms VOT as standard and +15 ms VOT as deviant. In the *phonemic in Spanish* condition participants heard a stimulus token with −20 ms VOT as standard and +15 ms VOT as deviant. As predicted, an MMN was elicited for the phonemic in English condition when the participants were in an English language context, but not a Spanish language context. Likewise, an MMN was observed for the phonemic in Spanish condition in the Spanish language context, but not the English language context. The authors take these findings to suggest that language context can indeed affect pre-attentive auditory change detection. While the present study did not set out to investigate the role of language context, the results of Winkler et al. (1999), Winkler et al. (2003) and García-Sierra et al. (2012) do suggest that sounds that are non-contrastive in a listener's L1 may be perceived differently as a result of experience. Moreover, bilingual listeners may demonstrate flexibility in their perceptual abilities as a result of the language context.

In sum, listeners' perception of speech sounds is strongly and systematically constrained by the native language phonology, with the discriminability of pairs of phones being influenced by phonological status in the native language. This pattern of relative insensitivity to phone pairs which are allophones of a single phoneme category in the listener's native language is observed both in behavioral and neural responses. While these patterns of perception may be optimal for listeners when listening to their native language, such learned, early, and automatic insensitivity to L1 allophones may present challenges for L2 learners who are faced with the task of establishing a novel contrast among familiar pairs of target language phones. These findings prompt the question of whether and to what extent these patterns of perception can be overcome with experience. In particular, do L1 context-dependent allophones continue to play a role in L2 perception?

In this study we further investigate the acquisition of novel target language contrasts among L1 context-dependent allophones by L2 learners. We take advantage of the cross-linguistic differences in the mappings between the phones [d], [ð], and [ɾ] and their respective phoneme categories in English and Spanish. To this end, two experiments were conducted to investigate the representation and processing of three sound contrasts [d]-[ð], [d]-[ɾ], and [ð]-[ɾ] by three participant groups: English native speakers, Spanish native speakers, and advanced L1 Spanish late-learners of English.

We used an AX discrimination task as a behavioral measure of participants' sensitivity to various tokens of three nonword pairings [idi]-[iði], [idi]-[iɾi], and [iði]-[iɾi]. Following Boomershine et al. (2008) (among others), it was expected that the same phonetic contrast would be perceived more readily by listeners for whom the pair is phonemic in their native language than by listeners for whom the pair is allophonically related, and that this difference in sensitivity should be reflected in participants' d' scores. Thus, higher d' scores are expected for Spanish listeners than English listeners for the [idi]-[iɾi] contrast which is phonemic in Spanish, and allophonic in English, whereas English listeners were expected to outperform the Spanish listeners on the [idi]-[iði] pair which is phonemic in English and allophonic in Spanish. Finally, both native English and Spanish speakers were expected to demonstrate comparable sensitivity to the [iði]-[iɾi] control contrast which is phonemic in both languages. Of particular interest is the performance of the advanced L1 Spanish late-learners of English for the [idi]-[iði] contrast which is allophonic in the listeners' L1. If learners have overcome the learned insensitivity to the phonetic distinction between [idi]-[iði] and have established a novel contrast between /d/ and /ð/ in English, we expect no difference in their performance for this pair from the performance of the English speaker group. However, if learners have not yet established a novel target language contrast among L1 positional variants in perception, then we expect they may continue to have difficulty discriminating the pair.

Magnetoencepholographic (MEG) recordings were also used to measure the detailed time-course of brain activity in each of the three listener groups. By making a three-way comparison of pre-attentive processing to the three phones of interest by Spanish, English, and L2 listeners we can gain insight into the interlanguage phonological representations of the L2 learners. By using the presence of an MMN as an index of category identification, we will be able to show whether L2 learners represent the phones [d], [ð], and [ɾ] as English speakers or Spanish speakers do. If early auditory brain responses are shaped by the functional significance of the sound categories in the listeners' native language (Kazanina et al., 2006), then we should observe a different pattern of results as a function of listener group. A significant MMN response is expected for both Spanish and English listeners for the control contrast (i.e., [iði]-[iɾi]). For the English group, a MMN response is also expected for the phonemic pair [idi]-[iði], but not for the [idi]-[iɾi] pair, which is allophonic in the language. In contrast, an MMN should be observed for Spanish listeners for the [idi]-[iɾi] pair, but not for the allophonically related pair [idi]-[iði]. With respect to the performance of the advanced late learners of English, we expect that if they have acquired the English /d/-/ð/ contrast, they will show evidence of perceptual sensitivity in their pre-attentive brain response. However, if they have not yet acquired the target language contrast, we expect them to perform like the native Spanish speaker group.

## Materials and methods

### Participants

Three groups of participants were recruited to participate in these experiments for monetary compensation; 15 English native speakers (Female = 5, Male = 10, mean age = 22.3 years, range = 19–28), 15 Spanish native speakers (Female = 8, Male = 7, mean age = 34.7 years, range = 23–45), and 15 advanced L1 Spanish late-learners of English (Female = 8, Male = 7, mean age = 30.1 years, range = 24–38). The learner group had a mean age of exposure of 10.1 yrs (*SD* = 3.5), had lived in the US for 6.2 yrs on average (*SD* = 5) and had 8.6 yrs of formal training in English (*SD* = 4.7). All participants tested strongly right-handed according to the Edinburgh Handedness Inventory (Oldfield, 1971) and reported no history of hearing or neurological disorder. All participants were recruited from the University of Maryland, College Park and the surrounding area. English speaking participants and the majority of the Spanish speaking learners of English were undergraduate and graduate students who studied or worked at the University of Maryland campus. The Spanish speakers with little/no experience with English were recruited from a neighboring community with a large Spanish speaking population. This group was largely comprised of immigrants from Central America who had recently arrived to the area and continue to use Spanish as their primary mode of communication. They report having had little exposure to English aside from what is heard on TV and the radio^6^.

The proficiency of each of the listener groups was assessed by self report. Participants were asked to rate their abilities in the areas of speaking, listening, reading, and writing on a scale of 1–10 (where 1 = poor and 10 = excellent) in both Spanish and English. The English speaker means were 10 (*SD* = 0) speaking, 9.9 (*SD* = 0.3) listening, 9.9 (*SD* = 0.3) reading, 9.9 (*SD* = 0.3) writing in English and 1.7 (*SD* = 0.9) speaking, 1.9 (*SD* = 0.9) listening, 2.1 (*SD* = 1.3) reading, and 1.6 (*SD* = 1.1) writing in Spanish. The Spanish speaker means were 2.7 (*SD* = 2.1) speaking, 3.5 (*SD* = 2.4) listening, 3.5 (*SD* = 2.4) reading, and 2.7 (*SD* = 1.8) writing for English and 10 (*SD* = 0) speaking, 10 (*SD* = 0) listening, 9.9 (*SD* = 0.3) reading, and 10 (*SD* = 0) writing in Spanish. The mean ratings for the Learner group in English were 8.0 (*SD* = 1.3) speaking, 8.5 (*SD* = 1.1) listening, 9.1 (*SD* = 0.9) reading, and 8.1 (*SD* = 1.4) writing. The means of the Learner group in Spanish were 9.9 (*SD* = 0.4) speaking, 10 (*SD* = 0) listening, 10 (*SD* = 0) reading, and 9.8 (*SD* = 0.6) writing.

### Stimuli

Materials for our experiments consisted of 10 natural tokens of each of the following VCV sequences: [idi], [iði], [iɾi] spoken by a single female speaker of American English with phonetic training. Multiple instances of each stimulus type were recorded using a head-mounted microphone in a soundproof room. The vowel [i] was chosen for the vowel context because Spanish [i] and English [i] have the greatest perceived similarity by listeners of both groups (Flege et al., 1994). The resulting stimulus set did not result in words in either Spanish or English. Because the phones [d] and [ð] and [d] and [ɾ] are in complementary distribution in Spanish and English, respectively, it was not possible to find a context in which all three phones occur naturally. For this reason, it should be noted that the [idi] tokens may not sound particularly natural to either speaker group. All [idi] tokens were produced with care by a native English speaker with phonetic training so as to avoid flapping. Each was later inspected by two additional trained phoneticians to ensure that intervocalic [d] was not produced as [ɾ]. To ensure that any observed differences in the MMN response could only be attributed to differences in the consonant (as opposed to the preceding vowel), the initial [i] from each token was removed and replaced with an identical [i] recorded in a neutral context (i.e., [isi]). The ten best stimulus tokens of each type were chosen on the basis of their perceived naturalness to native speakers of Spanish and English to ensure that each stimulus token was perceived as acceptable by native speakers of both languages. All experimental stimuli were normalized for intensity using Praat (Boersma and Weenink, 2009) and were presented to participants at a comfortable listening level (~70 dB).

One challenge for this kind of design is ensuring that the tokens used are relatively natural exemplars across both languages. We examined a number of acoustic parameters to determine to what extent this was true of the current stimuli. The initial [i] of each token had a duration of 160 ms, intensity of 77 dB, F0 of 190 Hz, F1 of 359 Hz, F2 of 2897 Hz, and F3 of 3372 Hz. The initial [i] was cross-spliced with the natural consonant and final [i] productions. The files were matched from positive going zero-crossing to positive going zero-crossing. The final [i] tokens had a mean duration of 177 ms (*SD* = 20), intensity of 75 dB (*SD* = 1.8), F0 of 172 Hz (*SD* = 8), F1 of 350 Hz (*SD* = 14), F2 of 2826 Hz (*SD* = 72), and F3 of 3278 Hz (*SD* = 66). These formant values for initial and final [i] tokens fall within the range of values for female speakers of American English reported by Hillenbrand et al. (1995) (F0 = 227 Hz (*SD* = 24), range = 155–275 Hz; F1 = 437 Hz (*SD* = 41), range = 331–531 Hz; F2 = 2761 Hz (*SD* = 147), range = 2359–3049 Hz; F3 = 3372 Hz (*SD* = 237), range = 2958–3831 Hz)). The vowel duration reported by Hillenbrand et al. (1995) for [i] is longer (306 ms (*SD* = 46), range = 222–433 ms) than the duration of the [i] tokens reported here. However, this is expected given that their recordings were elicited in a h_d context. The formant values also match fairly closely the values reported by Quilis and Esgueva (1983) for Spanish [i] (F1 = 241 Hz (*SD* = 32), range = 202–324 Hz; F2 = 2839 Hz (*SD* = 237), range = 2349–3321 Hz; F3 = 3358 Hz (*SD* = 249), range = 2632–3726 Hz), with the exception that the Spanish [i] has a lower F1 than English [i]. The mean duration of the consonant segments of interest measured from the F2 offset of V1 to the onset of F1 of V2 were 76 ms (*SD* = 10, range = 63–96 ms) for [d], 78 ms (*SD* = 13, range = 59–99 ms) for [ð], and 41 ms (*SD* = 5, range = 34–51 ms) for [ɾ]. These values are comparable to those reported for English by Lavoie (2001) and Stathopoulos and Weismer (1983) for initial and medial non-prestressed /d/ (i.e., 70, 80 and 37, 41 ms, respectively). Our speaker's [ð] productions were on average longer than those reported Lavoie (2001) for initial and medial non-prestressed environments (57 and 48 ms). For Mexican Spanish, Lavoie (2001) reports durations of 51, 24, and 55 ms for medial non-prestressed /d/ and /ɾ/ and initial non-prestressed /d/, respectively. The mean duration of the stimulus tokens measured from word onset to word offset from the Praat waveform was 426 ms (*SD* = 29, range = 384–480 ms) for [idi], 416 ms (*SD* = 9, range = 402–429 ms) for [iði], and 363 ms (*SD* = 20, range = 319–397 ms) for [iɾi]. Following Carrasco et al. (2012), we also computed a ratio of the minimum intensity of the consonant/maximum intensity of the following vowel as a measure of the relative intensity/degree of constriction of the consonant productions. A ratio that is close to one indicates a more open vowel-like production of the consonant, and a ratio that is closer to zero indicates a more stop-like realization. The ratio for the [d] was 0.70 (*SD* = 0.02), for [ð] was 0.77 (*SD* = 0.04) and for [ɾ] was 0.81 (*SD* = 0.03). While the ratios shouldn't be compared directly to those reported in Carrasco et al. (2012), since vowel contexts are known to affect these measures (Simonet et al., 2012)^7^ and the vowel contexts differ from those used in their study, what is worth noting is that the most vowel like production is the [ɾ] and the least vowel-like production is the [d]. The [ð] lies in between those two.

### Post-study identification task

To ensure that participants in the study also identified the stimuli as instances of the intended category, each performed a brief identification task following the MEG recording and the AX discrimination task. Participants were presented with 40 stimuli (each of the 30 experimental items and 10 filler items) and were instructed to use the keys 1, 2, and 3 to identify the stimulus they heard. Naturally, the labels for the identification task had to vary across language, such that the English speakers were asked to label stimuli as an instance of a nonword “eithee,” “eady,” or “other” and the Spanish speakers as the nonwords “idi,” “iri,” or “other.” In order to implement the task in a similar way across groups we had to decide which labeling to request from the Learners. Given that our primary interest in the identification task was to learn if our stimulus tokens would be categorized as instances of the expected stimulus type in the listeners' L1, we opted to use L1 labeling options for all three listener groups.

**Figure 2:**
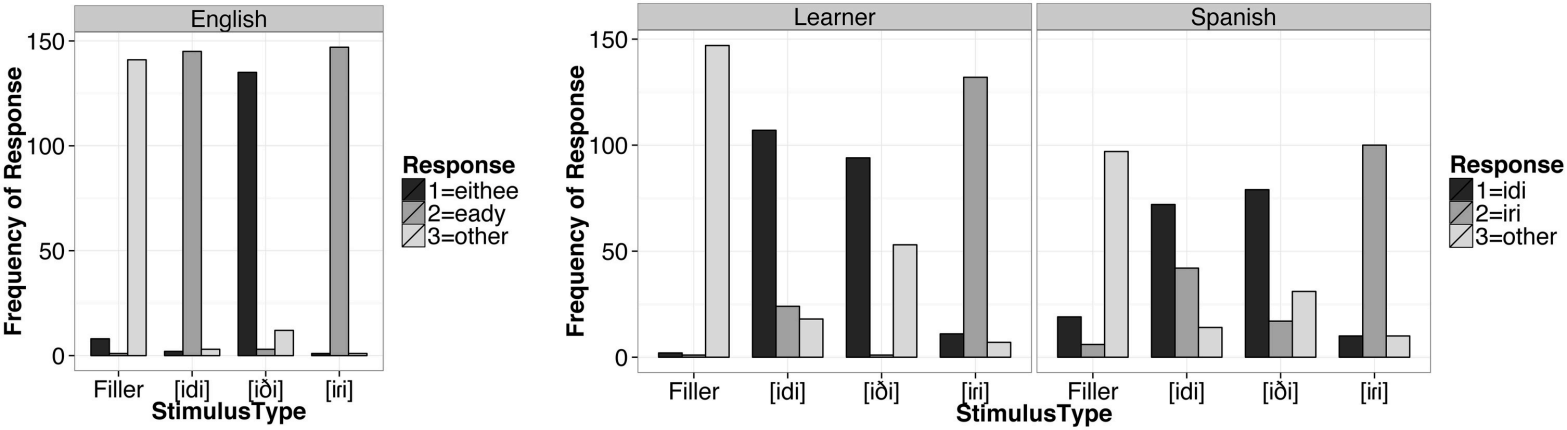
**Frequency of response label by stimulus type and listener group**.

Figure 2 shows the frequency of each response by stimulus type for each of the three language groups. All participants chose “other” predominantly for the filler items. English listeners chose “eithee” predominantly for the [iði] tokens, and “eady” for both the [idi] and [iɾi] tokens. Both Spanish listeners and the Learner group primarily chose “idi” as the label for [idi] and [iði] tokens, whereas [iɾi] tokens were predominantly identified as “iri” by Spanish listeners. Thus, the stimulus tokens used in the study can be heard as instances of the expected stimulus type in the listeners' L1 (at least on a conscious-labeling task).

## Procedures

### AX discrimination task

During the AX discrimination task participants wore headphones and were seated in a quiet room in front of a computer. The presentation of experimental stimuli was controlled by DMDX (Forster and Forster, 2003). In the AX discrimination task participants were presented two of the experimental stimuli which were either different tokens of the same nonword (i.e., [idi]-[idi], [iði]-[iði], [iɾi]-[iɾi]) or one of the six possible ordered pairings of different nonwords (i.e., [idi]-[iɾi], [idi]-[iði], [iði]-[iɾi], [iði]-[idi], [iɾi]-[iði], [iɾi]-[idi]). Participants responded to 32 same (16 AA, 16 BB) and 32 different (16 AB, 16 BA) trials per contrast, for a total of 192 test trials. Each stimulus was presented with an interstimulus interval (ISI) of 500 ms. Participants were instructed to press the “F” key on the keyboard with their left index finger if the two stimuli were two pronunciations of the same “word” and to press the “J” key with their right index finger if the paired stimuli corresponded to two different “words.” Participants were asked to respond as quickly and accurately as possible and had a maximum of 4 s to respond on each trial. Written instructions were provided in the native language of each listener group, as well as orally by the experimenter. Six practice trials without feedback preceded the test trials to ensure that participants understood and were comfortable performing the experimental task. These practice trials were repeated a second time in the case that participants still appeared uncertain about the task or uncomfortable with providing their response on the computer keyboard. The AX discrimination task lasted approximately 15 min and was divided into four blocks of 48 items with three self-timed breaks between each block.

### MEG recordings

Magnetic fields were recorded in DC (no high-pass filter) using a whole-head MEG device with 157 axial gradiometers (Kanazawa Institute of Technology, Kanazawa, Japan) at a sampling rate of 1 kHz. An online low pass filter of 200 and a 60 Hz notch filter were applied during data acquisition. All stimuli were presented binaurally via Etymotic ER3A insert earphones at a comfortable listening level (~70 dB). MEG recording sessions included 4 runs: 1 screening run and 3 experimental blocks which are described in greater detail below. Participants passively viewed a silent movie during the experimental runs to avoid fatigue. Each MEG recording session lasted approximately 90 min in total.

In the screening run, participants were presented approximately 100 repetitions of a 1 kHz sinusoidal tone. Each tone was separated by a randomly chosen ISI of 1000, 1400, or 1800 ms. Data from the screening run were averaged and examined to verify a canonical M100 response. The M100 is an evoked response which is produced whenever an auditory stimulus has a clear onset and is observed regardless of attentional state (Näätänen and Picton, 1987). Data from 45 participants run across the three participant groups showed a reliable bilateral M100 response with a source/sink reversal between anterior and posterior channels in the left and right hemisphere. Three additional participants were recruited and run on the screening task, but were excluded because they did not show a strong bilateral M100 response elicited by a 1-kHz pure tone at pretest. The M100 response elicited to non-speech tone stimuli were additionally used to select the auditory channels of interest for each of our participants for the MMN amplitude analysis.

**Figure 3:**
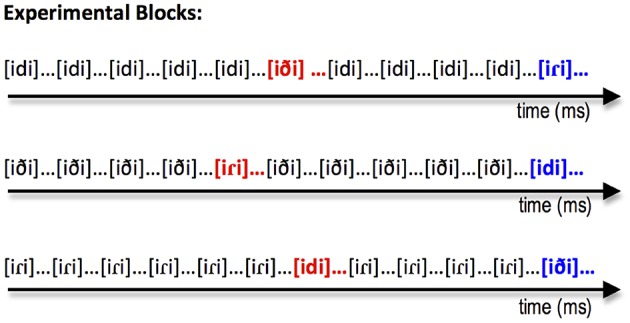
**Illustration of the structure of each of the three experimental blocks in our modified passive oddball paradigm**. Stimuli shown in black correspond to the stimulus type that served as the standard for that experimental block. The two types of deviants for a particular block are shown in red and blue.

In the experimental blocks, stimuli were presented using a modified version of the optimal passive oddball paradigm (Näätänen et al., 2004). In each of the three experimental blocks one of the three stimulus types (i.e., [idi], [iði], or [iɾi]) was presented frequently (i.e., the standard) and was followed by infrequent stimuli of the other two types. For example, in Figure 3, the first block shows [idi] as the frequent standard and [iði] and [iɾi] as the less frequent intervening deviant stimulus types. Following Phillips et al. (2000), there was no acoustic standard. Instead, participants were presented multiple non-orthogonally varying tokens from each category. This was done to avoid a purely acoustic interpretation of the elicited responses. Thus, the presence of an MMN serves as a measure of grouping of different acoustic tokens into phoneme or allophone categories. Each block consisted of 882 standards and 168 deviants (84 of each deviant type). A deviant was presented after a minimum of 4 and a maximum of 6 standards with the probability of deviant (either deviant type A or B) = 0.167. Each stimulus token was separated by an ISI that varied randomly between 600 and 1000 ms. Each of the three experimental blocks lasted approximately 20 min. Participants were given a short break after each 10 min of recording. Block order was counterbalanced across participants. Figure 3 shows the structure of each of the three blocks.

The experimental procedures were completed in the following order for all participants: [1] participants were provided an overview of the procedures and provided their informed consent, [2] participants completed a language background and handedness questionnaire to ensure they met the study requirements, [3] MEG recordings were made, and [4] AX discrimination and identification data were collected.

## Data analysis

### AX discrimination data

**Figure 4:**
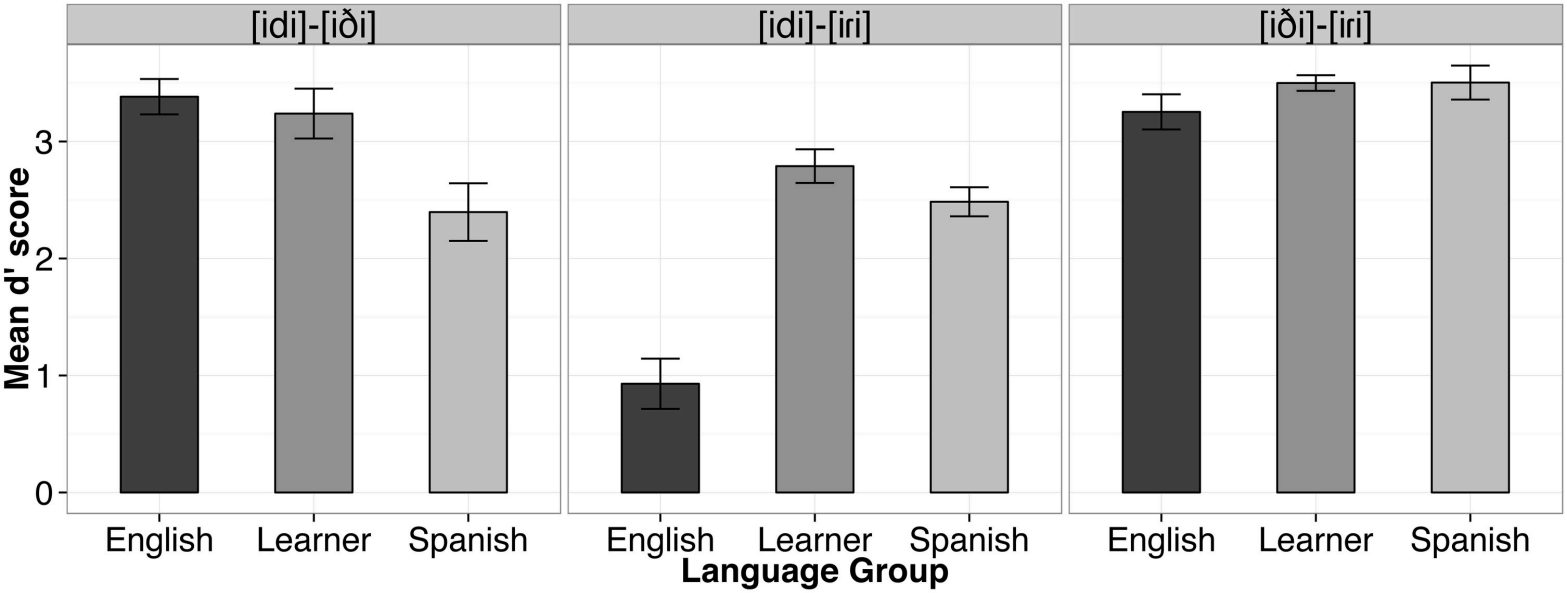
**Mean d' scores by language group and contrast**. Error bars represent one standard error of the mean.

Data from four Spanish participants (S003, S004, S011, S014) whose performance was at or below chance (i.e., 50% accuracy) on the control contrast (i.e., [iði]-[iɾi]) were excluded from subsequent AX discrimination analyses. For the remaining participants, d' scores were computed for each individual and each different pair according to the Same-Different Independent Observations Model (Macmillan and Creelman, 2005) using the dprime.SD() function from the *psyphy* package in R (Knoblauch, 2007). The result is a measure of sensitivity which factors out participants' response bias. The “hit rate” was computed as the proportion of “different" responses when the words in the pair were different. The “false alarm rate” was the proportion of “different" responses when the words in the pair were the same. To correct for extreme proportions (i.e., hit rates and false alarm rates of 0 or 1), we applied Laplace smoothing (Jurafsky and Martin, 2009). In probability theory, Laplace's Rule of Succession is used to estimate underlying probabilities when there are few observations, or for events that have not been observed to occur at all in some finite sample of data. The rule states that if we repeat an experiment that we know can result in a success or failure (in our case hit or false alarm), *n* times independently, and observe *s* successes, then the probability of success on the next repetition of the experiment is (*s*+1)∕(*n*+2). Thus, our best estimate of a participant's hit rate when 32 hits and 0 misses are observed across 32 different trials is (32+1)∕(32+2) (or 0.97). For a participant with a false alarm rate of 0, our best estimate of the false alarm rate is (0 + 1)∕(32 + 2) (or 0.03). As a result, the largest d' score that may be observed given our experimental materials with 32 different trials was 4.34. The d' values obtained for each test pair per subject ranged from 4.34 to 0. Two participants achieved the maximum d' score for one of the conditions (E015 for [iði]-[iɾi] and L009 for [idi]-[iði]). Figure 4 shows the mean d' score by language group and contrast. These d' scores were subsequently analyzed using linear mixed effects modeling.

### MEG data pre-processing

MEG data were imported into Matlab and de-noised using a multi-shift PCA noise reduction algorithm (de Cheveigné and Simon, 2007, 2008). Epochs included 100 ms pre-stimulus onset to 800 ms post-stimulus onset. Artifact rejection was conducted manually in MEG160 to exclude trials containing muscle and eye-related artifacts. All epochs were then averaged, baseline corrected over a 100 ms pre-stimulus interval, and filtered using a 0.03 to 30-Hz band-pass filter.

For each participant, the 10 strongest left hemisphere channels (5 from left anterior, 5 from left posterior) were identified and selected visually in MEG160 from the peak of the average M100 response to 1 kHz tones elicited during the auditory localizer pre-screening test. Because the MMNm to phoneme prototypes has been found to be stronger in the left hemisphere than in the right (Näätänen et al., 1997), we calculated the root mean square (RMS) amplitude of the MEG temporal waveforms over the left hemisphere channels selected on the basis of the pre-screening test. Trials were averaged separately for each participant and for each condition (i.e., three standard and six deviant types).

**Figure 5:**
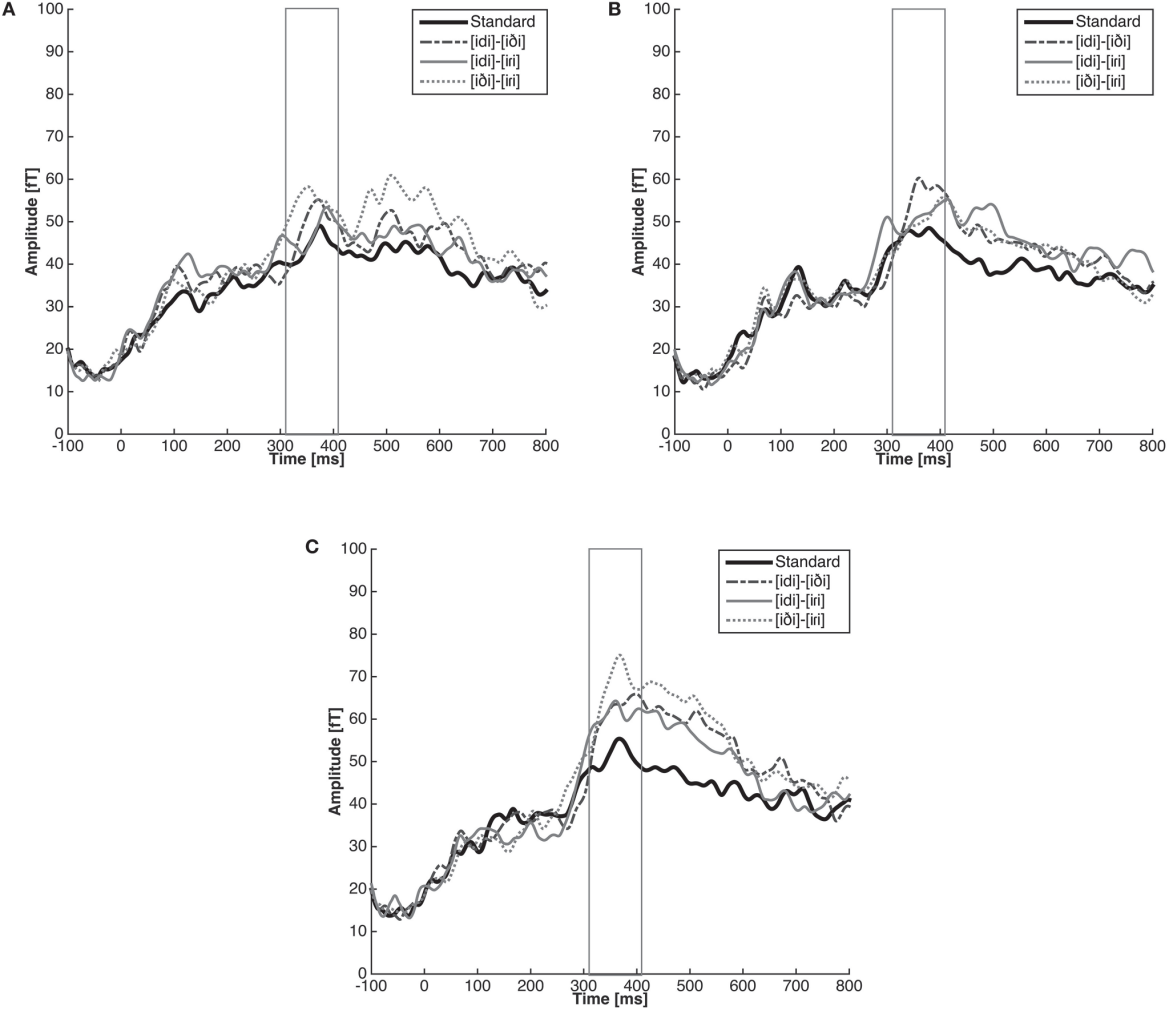
**Grand average RMS amplitude of the response by listener group (English (A), Spanish (B), and Learner (C)) and contrast ([idi]-[iDi], [idi]-[iRi], and [iDi]-[iRi])**. The response to each pair represents the summary deviant response for each of the three contrasts (A as standard with B as deviant and vice versa). The solid black line in each figure represents the mean RMS amplitude [fT] to pooled standards.

We created a single summary deviant response for each of the three contrasts by averaging together the two relevant deviant responses. For example, for the [iɾi]-[iði] control contrast, we averaged together the response to [iði] deviants in an [iɾi] block and the response to [iɾi] deviants in an [iði] block. The averaged responses elicited by standards were also pooled, resulting in a single summary standard response. The grand average waveform from −100 ms pre-stimulus to 800 ms post-stimulus was then computed for language group by averaging across participants (*n* = 15 per group) for each condition (i.e., [idi]-[iði], [idi]-[iɾi], [iði]-[iɾi], and Standard). These are shown in Figure 5. Although in this analysis we collapse across data from both directions of a given contrast (A as standard with B as deviant and vice versa), it is worth noting that in certain cases such as phonological underspecification, directionality impacts the size of the MMN response (Eulitz and Lahiri, 2004). In the current case, we had no a priori reason to expect a systematic impact of directionality and therefore we collapsed across directions to ensure sufficient power. However, for the interested reader we include a supplementary analysis of the MMN data separated by direction in the Supplementary Materials.

The mean RMS power over a single 100 ms time window from 310 to 410 ms for each of the participants for each of the experimental conditions was computed. This time window was chosen because the vowel offset and consonant onset occurred at 160 ms and the MMN is expected to occur about 150–250 ms following the onset of a detectable change. Our statistical comparisons used linear mixed effects modeling to examine whether the difference in the mean RMS of the response to deviants and the response to standards reached significance over the MMN time window (310–410 ms).

## Results

### d' scores

Statistical analyses of d' scores were performed with linear mixed effects modeling using R package *lme4* (Bates et al., 2015) with factors Language Group (English, Learner, Spanish), Contrast ([idi]-[iɾi], [idi]-[iði], [iði]-[iɾi]), and the Language Group × Contrast interaction as fixed effects and subject as a random effect in order to account for inter-subject variability. *P*-values were computed using the Satterthwaite's approximation for denominator degrees of freedom with the *lmerTest* package (Kuznetsova et al., 2014). We observed a main effect of Language Group [*F*_(2, 37)_ = 10.07, *p* < 0.001], and of Contrast [*F*_(2, 74)_ = 54.40, *p* < 0.001], as well as a Language Group by Contrast interaction [*F*_(4, 74)_ = 19.20, *p* < 0.001].

We conducted nine planned tests of our experimental hypotheses regarding listeners' sensitivity to allophonic vs. phonemic contrasts using simultaneous tests for general linear hypotheses with the *multcomp* package in R (Hothorn et al., 2008). *P*-values were adjusted using the single-step method. First, it was hypothesized that our three listener groups should not differ in performance on the control contrast (i.e., [iði]-[iɾi]), as the contrast is phonemic in both Spanish and English. This prediction was borne out. English listeners did not differ from Spanish listeners for this contrast (β = 0.25, *SE* = 0.25, *z* = 1.02, *p* = 0.91), nor did the d' scores of the English group and the Learner group (β = 0.25, *SE* = 0.23, *z* = 1.07, *p* = 0.89) or the Spanish group and the Learner group differ for this contrast (β = −0.003, *SE* = 0.25, *z* = −0.02, *p* = 1.00).

For the [d]-[ɾ] contrast, which is phonemic in Spanish, but allophonic in English, it was expected that the L1 Spanish listeners would outperform the English listeners. This prediction was also borne out. The English listeners performed significantly worse than both the Spanish listeners (β = 1.56, *SE* = 0.25, *z* = 6.31, *p* < 0.001) and the Learner group (β = 1.86, *SE* = 0.23, *z* = 8.06, *p* < 0.001), supporting the hypothesis that phonological status influences perception on our AX discrimination task. No difference was observed between the Spanish and Learner group for this contrast (β = 0.30, *SE* = 0.25, *z* = 1.22, *p* = 0.82).

For us the most important question is what level of discrimination performance Spanish late-learners of English would show on a contrast that is phonemic in English but allophonic in Spanish (i.e., [d]-[ð]). First, as expected, the Spanish group performed significantly poorer on this contrast than the English listeners (β = −0.99, *SE* = 0.25, *z* = −4.00, *p* < 0.001), again providing support for differential processing of the contrast as a function of phonemic status in the language. Interestingly, with respect to our primary research question, a significant difference was observed for the L1-allophonic contrast [d]-[ð] for the Spanish and Learner listener groups (β = 0.84, *SE* = 0.25, *z* = 3.37, *p* < 0.01), with larger d' scores observed for the Learners than Spanish listeners. Moreover, no significant difference in d' was observed between the English listener group and the Learner group (β = −0.14, *SE* = 0.23, *z* = −0.63, *p* = 0.99), suggesting that the participants in the Learner group may have acquired a target language contrast among the phones [d]-[ð] which function as context-dependent allophones in their L1.

### Mean RMS amplitude of MMN

We again used linear mixed effects modeling in R to conduct the statistical analyses of mean RMS amplitude over the 310–410 ms time window. Our first linear mixed effects analysis was designed to confirm that there were no reliable differences between the responses to the different standards. This is important to establish because we would like to collapse across the response to standards in our subsequent critical planned comparisons of the MMN response by contrast. Analyses of mean RMS amplitude for the response elicited by the standards consisted of fixed effects Language Group (English, Learner, Spanish) and Standard Type ([idi] standard, [iði] standard, [iɾi] standard), as well as Language Group × Standard Type interaction and subject as random effect. These statistical analyses revealed no significant results, suggesting that the mean power elicited by standard stimuli did not differ by Language Group [*F*_(2, 42)_ = 0.43, *p* = 0.66] or Standard Type [*F*_(2, 84)_ = 2.12, *p* = 0.13], nor did these factors interact [*F*_(4, 84)_ = 0.13, *p* = 0.96]. We take this to suggest that listeners are able to form a coherent representation for the standard stimuli and that we are justified in comparing responses elicited by deviants against pooled standards.

**Figure 6:**
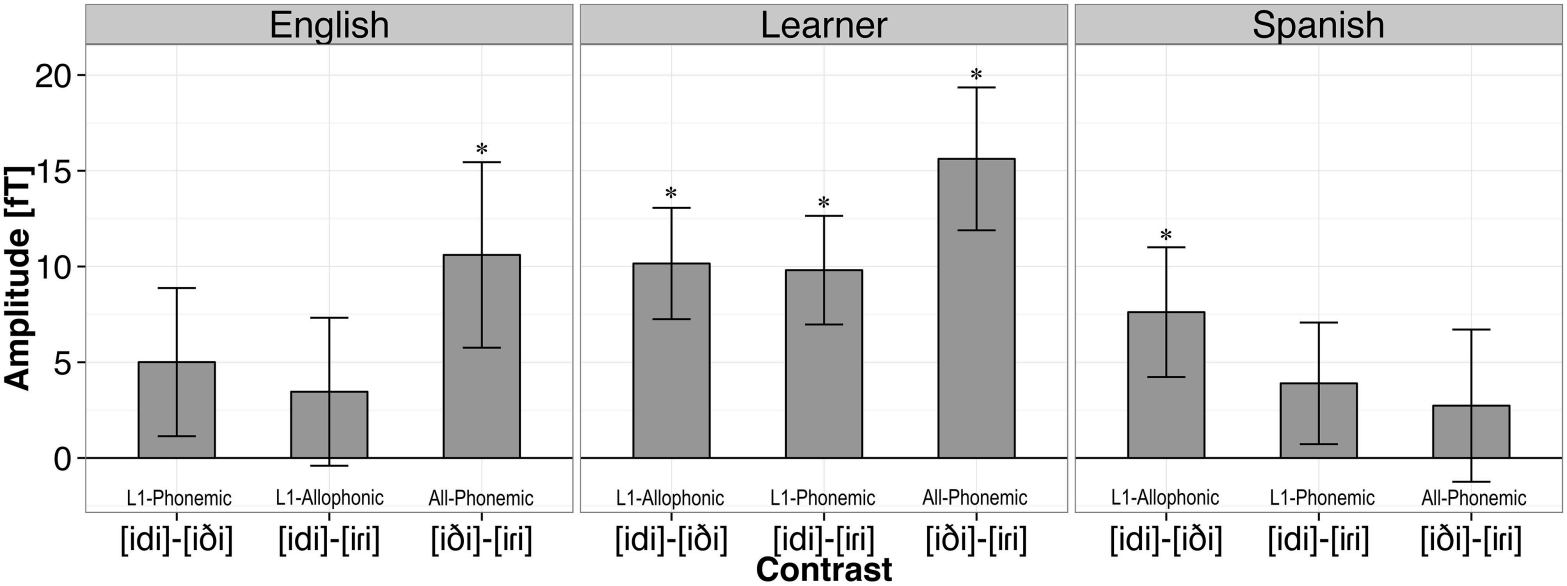
**Mean RMS amplitude of the MMN [fT] by language group and contrast**. Each bar represents the difference between the summary deviant response (A as standard with B as deviant and vice versa) and the response to the pooled standards. Error bars represent one standard error of the mean. Asterisks indicate significant MMN responses.

Figure 6 shows the mean RMS amplitude of the MMN for each of the three contrasts for each listener group. Analyses of the MMN amplitude consisted of fixed effects Language Group (English, Learner, Spanish) and Stimulus Type (Allophonic, Phonemic, Control, Standard), as well as Language Group × Stimulus Type interaction and subject as random effect. There was no main effect of Language Group [*F*_(2, 42.14)_ = 1.01, *p* = 0.37]. However, the main effect of Stimulus Type reached significance [*F*_(3, 351)_ = 7.21, *p* < 0.001]. No interaction between Language Group and Stimulus Type was observed [*F*_(6, 351)_ = 1.32, *p* = 0.25].

In our statistical analyses of the listeners' responses to deviants, we conducted three planned comparisons separately for each listener group using simultaneous tests for general linear hypotheses with the *multcomp* package in R (Hothorn et al., 2008). *P*-values were adjusted using the single-step method. We compared each groups' response to the pooled standard (i.e., responses to the stimuli [idi], [iði], and [iɾi] when they are presented as standards in a block) to the groups' responses to the summary deviant response for each of the three contrasts.

As expected for the English listeners, the response to the control contrast [iði]-[iɾi] was larger than the response to the standard stimuli (β = 21.21, *SE* = 8.18, *z* = 2.59, *p* < 0.05). Again as expected, we found no difference between the magnitude of the response elicited by the standards and the allophonic pair [idi]-[iɾi] (β = 6.92, *SE* = 8.18, *z* = 0.85, *p* = 0.75). Unexpectedly, we found no difference between the response to the standard and the response to the English phonemic contrast [idi]-[iði] (β = 10.02, *SE* = 8.18, *z* = 1.22, *p* = 0.49).

Unfortunately, the MMN responses for the Spanish listeners followed none of our predictions. We found a marginal difference between the response to the standard stimuli and the [idi]-[iði] pair which are phonologically related in the language (β = 15.23, *SE* = 6.51, *z* = 2.34, *p* = 0.05). We also found no difference between the standards and either the [iði]-[iɾi] control pair (β = 5.47, *SE* = 6.51, *z* = 0.84, *p* = 0.76) or the phonemic [idi]-[iɾi] pair (β = 7.80, *SE* = 6.51, *z* = 1.20, *p* = 0.51).

For the critical learner group, the MMN results followed the pattern predicted according to the hypothesis that learners successfully implemented the phonological knowledge of their second language at an early, pre-attentive stage of processing. A significant difference was observed between the standards and L1 allophonic contrast [idi]-[iði] (β = 20.31, *SE* = 8.19, *z* = 2.48, *p* < 0.05), the phonemic contrast [idi]-[iɾi] (β = 19.62, *SE* = 8.19, *z* = 2.40, *p* = 0.05), and the control contrast [iði]-[iɾi] (β = 31.24, *SE* = 8.19, *z* = 3.82, *p* < 0.001). These results suggest that the learners' ability to distinguish the contrasts that were observed in the behavioral data is also apparent at the stage of early pre-attentive processing, regardless of the pairs phonological status in the L1.

## Discussion

In this study we explored the impact of phonological knowledge on perceptual categorization, particularly in cases in which the phonemic status in a late-learned second language directly conflicts with the native language. Our Spanish and English listeners demonstrated greater sensitivity for nonword pairs distinguished by phonemic than by allophonic contrasts on an AX discrimination task, mirroring previous findings. Interestingly, Spanish late-learners demonstrated sensitivity (large d' scores and MMN responses) to all three contrasts, suggesting that these L2 learners may have established a novel [d]-[ð] contrast despite the phonological relatedness of these sounds in the L1. We discuss each of these findings in turn.

### Phoneme-based equivalence classes in the L1

Our behavioral findings from the native speaker groups provide support for the hypothesis that listeners form equivalence classes on the basis of phoneme categories. In particular, we observed better discrimination of the [idi]-[iɾi] contrast by Spanish listeners for whom the pair are phonemic than by English listeners for whom the pair is allophonic in their L1. Similarly, English listeners outperformed Spanish listeners in the discrimination of the [idi]-[iði] pair which is phonemic in English, but allophonic in Spanish. Finally, both Spanish and English listener groups performed comparably well on the [iði]-[iɾi] control contrast which is a phonemic distinction in both languages. These results replicate previous behavioral findings from Boomershine et al. (2008), and provide additional evidence that phonological relatedness among sounds reduces their perceptual similarity in native listeners.

The MEG data also provides partial support for the hypothesis that listeners establish equivalence classes on the basis of phonemes. Given this hypothesis, we expected to observe an MMN when the stimulus presented as the deviant is in contrast in the listener's native language with the stimulus serving as the standard in an experimental block, but not when the standard and deviant are phonologically related as allophones of the same phoneme in the listeners' L1. As expected, a significant MMN was observed for the [iði]-[iɾi] control contrast, but not for the allophonic [idi]-[iɾi] contrast for English listeners. Contrary to our expectations, however, no MMN was observed for the phonemic [idi]-[iði] pair. In contrast with the data from the English listeners, the results for the Spanish listeners did not provide support for our hypothesis. A significant MMN was observed for the [idi]-[iði] contrast, which is allophonic in Spanish, while no MMN was observed for either the [idi]-[iri] or the [iði]-[iɾi] pair which are phonemic in Spanish.

It is not clear how to explain the unexpected MMN patterns observed in the two native listener groups. First, any explanation based on poor stimulus quality seems inconsistent with the behavioral data, which showed the predicted pattern of discrimination across groups for all contrasts (although it is of course logically possible that the behavioral responses were based on a late-stage process that the early MMN does not reflect). Second, it is not clear how any simple explanation based on the acoustic properties of the stimuli could explain the cross-linguistic differences in responses. However, we note that the only surprising datapoint in the English listener data was the absence of a significant MMN in the phonemic [idi]-[iði] contrast, but that the response was trending in the right direction. Therefore, we might speculatively attribute this result to a Type II error.

One factor that may have reduced our power to detect MMN differences in the current paradigm is that the position of the deviant within the standard stream was somewhat more predictable than in many MMN studies. In our experiment, a deviant always occurred after either 4, 5, or 6 intervening standards. Previous work has demonstrated that when the position of a deviant within the standard stream is completely predictable, the MMN is almost completely neutralized (see Sussman et al., 2014 for review) and therefore the partial predictability may have reduced the overall strength of MMN effects. Although in the current case this increase in predictability was partly driven by our desire to examine three different contrasts (forcing a smaller standard to deviant ratio with fewer trials between deviants), in future work it would be useful to investigate the same contrast with greater unpredictability.

In addition, the slightly non-canonical status of the speech stimuli as neither perfectly English-like nor perfectly Spanish-like may have caused some of the unexpected MMN patterns observed in the Spanish and English groups. In an active task like AX discrimination, increased attention might mitigate the impact of slightly non-canonical tokens on categorization, but in a passive listening mode, as in the MMN paradigm, participants might not have automatically perceived and grouped the tokens according to their native speech categories. On the other hand, the bilingual participants might be more permissive of irregularities even in passive listening, based on their exposure to different distributions of sounds across the two languages. Strange and Shafer (2008), in their Automatic Selective Perception (ASP) model, have suggested that the perception of nonnative contrasts is dependent on task demands that determine the degree of attentional focus that is placed on the phonetic details of the stimuli. In support of the model, Hisagi et al. (2010) demonstrated that selective attention enhanced the magnitude of the MMN responses of the American English listeners to the nonnative Japanese vowel length contrast. Attention may likewise be required for listeners to categorize familiar phonological contrasts when the contrasts are specified by slightly different acoustic-phonetic parameters. Future work could examine the potential role of attention by manipulating attention directly and by incorporating active tasks which allow the researcher to monitor the participants' focus of attention. We believe that an additional related factor in the unexpected MMN pattern for Spanish and English speaker groups may be the fact that the [idi] token does not match prior language experience for either Spanish or English speakers, as [d] does not occur intervocalically in either language. Addressing either of these factors in future work will be challenging however, because it is not possible to create tokens for the full set of contrasts that are fully and equally natural tokens of Spanish and English, and as allophonic variation is context dependent, contrasting allophones in the MMN design necessarily requires one of the allophones to be presented in an unnatural context.

### Acquiring new mappings among familiar phones

Our primary research question asked whether advanced L1 Spanish late-learners of English overcome learned insensitivities to L1 context-dependent allophones and acquire a new target-language contrast among familiar phones [d] and [ð]. The behavioral and neural data from L2 learners which we report here converge to suggest that the answer to this question is affirmative. On both tasks we observed no difference between learners' ability to discriminate between phone pairs which are L1 allophones and L1 phonemes, suggesting that they do not classify the two phones as allophones of the same underlying phoneme category. That is, with experience, the advanced L2 learners in our study have acquired adequate knowledge of the L2 phonological system to distinguish the English /d/-/ð/ contrast in perception. Moreover, this learned sensitivity is observable both behaviorally, and in listeners' early, pre-attentive brain responses. We note that the neural data must be interpreted somewhat more cautiously than the behavioral data. Although the MMN pattern observed in the late-learner group was exactly what was predicted if they had successfully acquired the L2 phonological system, the two native listener groups did not show the MMN patterns predicted based on their L1 phonology, as described above. Therefore, further replication will be needed to confirm the interpretation of the MMN pattern in the late-learner group.

Given that our behavioral and MEG data from our Learner group was elicited in an English language context (all testing was conducted in an English speaking environment and all interactions and instructions were given in English), we might have expected the Learners' neural and behavioral responses to look maximally English-like (i.e., discriminating [d]-[ð] and [ð]-[ɾ], but not [d]-[ɾ]). However, this was not what was observed (contra García-Sierra et al., 2012 and in line with Winkler et al., 2003). We note, however, that we did not actively attempt to manipulate language context in our study. It is possible that Learners' performance on [d]-[ɾ] would have been different had we done so. It is also possible that other factors contribute to the observed effects, such as the language dominance of the participants or the proportion of L1/L2 use. These interesting possibilities should be taken up in future research.

A question that arises naturally from our learner data is: how do L2 learners acquire the ability to perceive novel target language contrasts among familiar phones? In particular, what is the role of the input in shaping the learners' hypotheses about the phonological system they are acquiring, and how do learners' expectations about the characteristics of the target language influence the learning process. With respect to mechanisms, three possibilities have been discussed in the infant literature (Seidl and Cristia, 2012, provide a more detailed review). First, it has been proposed that some information about phonological status may be available in the acoustic signal. That is, allophones may be more acoustically similar than phonemes. Some support for the plausibility of a phonetic mechanism comes from acoustic analyses of nasal and oral vowel allophones and phonemes in corpora of infant directed speech (Seidl et al., 2014). However, more research is needed to demonstrate the extent to which reliable information of this sort is available in the input to learners and to investigate whether infants and adults actually can and do use this information when learning about phonological status.

Another possibility is that listeners' make use of distributional information (Maye et al., 2002), such as the phonological context in which phones occur to learn phonological status. It is well established that both infants and adults can track the distribution of phones in acoustic space (Maye and Gerken, 2000; Hayes-Harb, 2007) and other phonological units, such as syllables (Saffran et al., 1996). Distribution-based learning mechanisms are also assumed to play a role in infants learning of allophones (Peperkamp et al., 2003, 2006; White et al., 2008), and have been invoked in the acquisition of L2 allophonic alternations (Shea and Curtin, 2010, 2011; Shea, 2014). In such cases, L2 learners are thought to acquire knowledge about the phonological patterning of L2 allophonic variants by tracking the distribution of target language phones with respect to their conditioning contexts. In the case of allophonic splits, it would seem that distributional learning is also required. Learners must learn that phones which are contextually licensed in their L1 are not restricted to the same phonological environments in their L2. For example, [ð] is permitted word-initially and word-finally in English, in addition to in word-medial post-vocalic non-prestress environments as in Spanish. More work is needed to investigate how the input is processed by adult L2 learners and to demonstrate that novel contrasts among L1 context-dependent allophones can indeed be learned by tracking phones and their respective phonological contexts.

Lexical mechanisms of various sorts have also been proposed, such as knowledge of word meanings and knowledge of words' phonological forms. For example, the availability of minimal pairs has been shown to enhance the perception of nonnative phonetic contrasts in both infants and adults (Hayes-Harb, 2007; Yeung and Werker, 2009). More recently, acquisition researchers have investigated the role of word contexts in phonetic category learning, demonstrating that infants and adults are sensitive to and can use distinct word forms (in the absence of visual referents or knowledge of word meanings) to constrain their interpretation of phonetic variability (Feldman et al., 2013).

Finally, in addition to the implicit learning mechanisms mentioned above, it has also been suggested that adults might avail themselves of explicit learning mechanisms and that these may serve to initiate the acquisition process (Shea, 2014). The effectiveness of various types of explicit input to L2 learners should be taken up in future research.

In sum, the behavioral and neural results presented here suggest that phonological relatedness influences perceived similarity, as evidenced by the results of the native speaker groups, but may not cause persistent difficulty for advanced L2 learners in perception. Instead, L2 learners overcome learned insensitivities to L1 allophones in perception as they gain experience with the target language. These findings provide a starting point to investigate when and how this learning takes place, as well as determine the respective contributions of the proposed mechanisms to the acquisition of novel target language contrasts among L1 context-dependent allophones in the L2.

## Author contributions

Conceived and designed the experiments: SB, AN, EL, NF, WI. Performed the data collection: SB, AN. Analyzed the data: SB, EL, WI. Wrote the manuscript: SB. Revised the manuscript critically for important intellectual content: SB, AN, EL, NF, WI. Provided final approval of the version to be published: SB, AN, EL, NF, WI. Agree to be accountable for all aspects of the work in ensuring that questions related to the accuracy or integrity of any part of the work are appropriately investigated and resolved: SB, AN, EL, NF, WI.

## Funding

This research was partially supported by a University of Utah University Research Committee Faculty Research and Creative Grant awarded to SB, as well as a University of Maryland, Department of Linguistics, Baggett Scholarship awarded to AN.

### Conflict of interest statement

The authors declare that the research was conducted in the absence of any commercial or financial relationships that could be construed as a potential conflict of interest.

## Footnotes

^1^Waltmunson (2005) reports that the intervocalic spirantization of /d/ occurs 99% of the time.

^2^Patterson and Connine (2001) report that the flapped variant occurs 94% of the time in its conditioning environment.

^3^It is is worth noting that to attain truly native-like competence in English, the Spanish learner must also learn to treat the phones [d] and [ɾ] as allophones of the same phoneme in English. Since this would involve the joining of L1 allophones, we might call this learning scenario ‘allophonic union.’

^4^Other related work in L2 phonology has investigated the acquisition of positional variants in the target language by L2 learners in production (Zampini, 1994; Shea and Curtin, 2011) and perception (Shea and Curtin, 2010; Shea and Renauld, 2014).

^5^It is worth noting that these results were observed despite the fact that [d] does not occur naturally in an intervocalic environment in either Spanish or English.

^6^It is worth noting that, in addition to language experience, the participants in the Spanish speaking group likely differ from the listeners in the other two groups in a number of other respects, including SES, level of education, experience and level of comfort working with computers, etc. While it may have been possible to find a better matched group of Spanish speakers elsewhere, we were constrained by location of accessible MEG equipment. This is not an obvious concern for our MEG data (which requires no behavioral response), but could impact the quality of our behavioral data which required participants to respond by pressing buttons on a computer keyboard.

^7^Simonet et al. (2012) report several continuous measurements of relative intensity. The authors argue that even among intervocalic tokens of /d/, the height of the preceding vowel conditions the degree of constriction of the consonant. Importantly, they report that in Iberian Spanish /d/ is more constricted after a high vowel than after a mid or low vowel.

## References

[B1] Bates D. Maechler M. Bolker B. Walker S. (2015). lme4: Linear Mixed-Effects Models Using Eigen and S4. R package version 1.1-1.10.

[B2] Best C. T. (1995). A direct realist perspective on cross-language speech perception, in Speech Perception and Linguistic Experience: Issues in Cross-Language Research, ed Strange W. (Timonium, MD: York Press), 171–204.

[B3] Best C. T. Tyler M. D. (2007). Nonnative and second-language speech perception: commonalities and complementarities, in Language Experience and Second Language Speech Learning: In Honor of James Emil Flege, Volume 17 of Language Learning and Language Teaching, eds Bohn O.-S. Munro M. J. (Philadelphia, PA: John Benjamins Publishing Company), 13–34.

[B4] Boersma P. Weenink D. (2009). Praat: Doing Phonetics by Computer [Computer Program]. Version 5.1.13.

[B5] Boomershine A. Currie Hall K. Hume E. Johnson K. (2008). The impact of allophony versus contrast on speech perception, in Contrast in Phonology: Theory, Perception, Acquisition, Volume 13 of Phonology & Phonetics, eds Avery P. Dresher B. E. Rice K. (Berlin: Mouton de Gruyter), 145–172.

[B6] Carrasco P. Hualde J. I. Simonet M. (2012). Dialectal differences in Spanish voiced obstruent allophony: Costa Rican versus Iberian Spanish. Phonetica 69, 149–179. 10.1159/000345199 23258464

[B7] de Cheveigné A. Simon J. Z. (2007). Denoising based on time-shift PCA. J. Neurosci. Methods 165, 297–305. 10.1016/j.jneumeth.2007.06.003 17624443 PMC2018742

[B8] de Cheveigné A. Simon J. Z. (2008). Sensor noise suppression. J. Neurosci. Methods 168, 195–202. 10.1016/j.jneumeth.2007.09.012 17963844 PMC2253211

[B9] Eckman F. R. Elreyes A. Iverson G. K. (2001). Allophonic splits in L2 phonology: the question of learnability. Int. J. English Stud. 1, 21–51.

[B10] Eckman F. R. Elreyes A. Iverson G. K. (2003). Some principles of second language phonology. Second Lang. Res. 19, 169–208. 10.1191/0267658303sr2190a

[B11] Escudero P. (2005). Linguistic Perception and Second Language Acquisition: Explaining the Attainment of Optimal Phonological Categorization. Ph.D. thesis, Utrecht University, Netherlands.

[B12] Eulitz C. Lahiri A. (2004). Neurobiological evidence for abstract phonological representations in the mental lexicon during speech recognition. J. Cogn. Neurosci. 16, 577–583. 10.1162/089892904323057308 15185677

[B13] Feldman N. H. Myers E. B. White K. S. Griffiths T. L. Morgan J. L. (2013). Word-level information influences phonetic learning in adults and infants. Cognition 127, 427–438. 10.1016/j.cognition.2013.02.007 23562941 PMC3646897

[B14] Flege J. E. (1995). Second language speech learning: theory, findings, and problems, in Speech Perception and Linguistic Experience: Issues in Cross-Language Research, ed Strange W. (Timonium, MD: York Press), 233–276.

[B15] Flege J. E. Munro M. J. Fox R. A. (1994). Auditory and categorical effects on cross-language vowel perception. J. Acoust. Soc. Am. 95, 3623–3641. 8046152 10.1121/1.409931

[B16] Forster K. I. Forster J. C. (2003). DMDX: a windows display program with millisecond accuracy. Behav. Res. Methods 35, 116–124. 10.3758/BF03195503 12723786

[B17] García-Sierra A. Ramírez-Esparza N. Silva-Pereyra J. Siard J. Champlin C. A. (2012). Assessing the double phonemic representation in bilingual speakers of Spanish and English: an electrophysiological study. Brain Lang. 121, 194–205. 10.1016/j.bandl.2012.03.008 22534571

[B18] Goto H. (1971). Auditory perception by normal Japanese adults with sounds “L” and “R. Neuropsychologia 9, 317–323. 5149302 10.1016/0028-3932(71)90027-3

[B19] Hammerly H. (1982). Contrastive phonology and error analysis. Int. Rev. Appl. Linguist. Lang. Teach. 20, 17–34.

[B20] Hardy J. E. (1993). Phonological learning and retention in second language acquisition, in Confluence: Lingusitics, L2 Acquisition and Speech Pathology, Volume 4 of Language Acquisition & Language Disorders, ed Eckman F. R. (Philadelphia, PA: John Benjamins Publishing Company), 235–247.

[B21] Harnsberger J. D. (2001). The perception of Malayalam nasal consonants by Marathi, Punjabi, Tamil, Oriya, Bengali, and American English listeners: a multidimensional scaling analysis. J. Phon. 29, 303–327. 10.1006/jpho.2001.0140

[B22] Hayes-Harb R. (2007). Lexical and statistical evidence in the acquisition of second language phonemes. Second Lang. Res. 23, 65–94. 10.1177/0267658307071601

[B23] Herd W. (2011). The Perception and Production Training of /d/, /R/, and /r/ in L2 Spanish: Behavioral, Psycholinguistic, and Neurolinguisitic Evidence. Ph.D. thesis, University of Kansas.

[B24] Hillenbrand J. Getty L. A. Clark M. J. Wheeler K. (1995). Acoustic characteristics of American English vowels. J. Acoust. Soc. Am. 97:3099. 10.1121/1.411872 7759650

[B25] Hisagi M. Shafer V. L. Strange W. Sussman E. S. (2010). Perception of a Japanese vowel length contrast by Japanese and American English listeners: behavioral and electrophysiological measures. Brain Res. 1360, 89–105. 10.1016/j.brainres.2010.08.092 20816759 PMC2994183

[B26] Hothorn T. Bretz F. Westfall P. (2008). Simultaneous inference in general parametric models. Biom. J. 50, 346–363. 10.1002/bimj.200810425 18481363

[B27] Iverson P. Kuhl P. K. Akahane-Yamada R. Diesch E. Tohkura Y. Kettermann A. (2003). A perceptual interference account of acquisition difficulties for nonnative phonemes. Cognition 83, B47–B57. 10.1016/S0010-0277(02)00198-1 12499111

[B28] Johnson K. Babel M. (2010). On the perceptual basis of distinctive features: evidence from the perception of fricatives by Dutch and English speakers. J. Phon. 38, 127–136. 10.1016/j.wocn.2009.11.001

[B29] Jurafsky D. Martin J. H. (2009). Speech and Language Processing: An Introduction to Natural Language Processing, Computational Linguistics, and Speech Recognition, Series In Artificial Intelligence, Pearson Education, Inc., 2nd Edn. Upper Saddle River, NJ: Prentice Hall.

[B30] Kazanina N. Phillips C. Idsardi W. J. (2006). The influence of meaning on the perception of speech sounds. Proc. Natl. Acad. Sci. U.S.A. 103, 11381–11386. 10.1073/pnas.0604821103 16849423 PMC3020137

[B31] Kenstowicz M. (1994). Phonology in Generative Grammar. Cambridge, MA: Blackwell Publishers.

[B32] Knoblauch K. (2007). Psyphy: Functions for Analyzing Psychophysical Data in R. R package version. 01–7.

[B33] Kuhl P. K. Williams K. A. Lacerda F. Stevens K. N. Lindblom B. (1992). Linguistic experience alters phonetic perception in infants by 6 months of age. Science 255, 606–608. 10.1126/science.1736364 1736364

[B34] Kuznetsova A. Bruun Brockhoff P. Haubo Bojesen Christensen R. (2014). lmerTest: Tests for Random and Fixed Effects for Linear Mixed Effect Models (lmer Objects of lme4 Package). R package version 2.0–11.

[B35] Lado R. (1957). Linguistics Across Cultures: Applied Linguistics for Language Teachers. Ann Arbor, MI: University of Michigan Press.

[B36] Lavoie L. M. (2001). Consonant Strength: Phonological Patterns and Phonetic Manifestations. New York, NY: Garland Publishing, Inc.

[B37] Lisker L. Abramson A. S. (1970). The voicing dimension: some experiments in comparative phonetics, in Proceedings of the sixth International Congress of Phonetic Sciences (Prague: Academia), 563–567.

[B38] Macmillan N. A. Creelman C. (2005). Detection Theory: A User's Guide, 2nd Edn. Mahwah, NJ: Lawrence Erlbaum Associates.

[B39] Maye J. Gerken L. (2000). Learning phonemes without minimal pairs, in Proceedings of the 24th Annual Boston University Conference on Language Development, Vol. 2, eds Howell S. C. Fish S. A. Keith-Lucas T. (Somerville, MA: Cascadilla Press), 522–533.

[B40] Maye J. Werker J. F. Gerken L. (2002). Infant sensitivity to distributional information can affect discrimination. Cognition 82, B101–B111. 10.1016/S0010-0277(01)00157-3 11747867

[B41] McLaughlin J. Osterhaut L. Kim A. (2004). Neural correlates of second-language word learning: minimal instruction produces rapid change. Nat. Neurosci. 7, 703–704. 10.1038/nn1264 15195094

[B42] Näätänen R. (1992). Attention and Brain Function. Hillsdale, NJ: Lawrence Erlbaum Associates.

[B43] Näätänen R. Lehtokoski A. Lennes M. Cheour M. Huotilainen M. Iivonen A.. (1997). Language-specific phoneme representations revealed by electric and magnetic brain responses. Nature 385, 432–434. 10.1038/385432a0 9009189

[B44] Näätänen R. Pakarinen S. Rinne T. Takegata R. (2004). The mismatch negativity (MMN): towards the optimal paradigm. Clin. Neurophysiol. 115, 140–144. 10.1016/j.clinph.2003.04.001 14706481

[B45] Näätänen R. Picton T. (1987). The N1 wave of the human electric and magnetic response to sound: a review and an analysis of the component structure. Psychophysiology 24, 375–425. 3615753 10.1111/j.1469-8986.1987.tb00311.x

[B46] Oldfield R. C. (1971). The assessment and analysis of handedness: the Edinburgh inventory. Neuropsychologia 9, 97–113. 5146491 10.1016/0028-3932(71)90067-4

[B47] Patterson D. Connine C. (2001). Variant frequency in flap production. Phonetica 58, 254–275. 10.1159/000046178 11641632

[B48] Pegg J. E. Werker J. F. (1997). Adult and infant perception of two English phones. J. Acoust. Soc. Am. 102, 3742–3753. 9407666 10.1121/1.420137

[B49] Peperkamp S. Le Calvez R. Nadal J.-P. Dupoux E. (2006). The acquisition of allophonic rules: statistical learning with linguistic constraints. Cognition 101, B31–B41. 10.1016/j.cognition.2005.10.006 16364279

[B50] Peperkamp S. Pettinato M. Dupoux E. (2003). Allophonic variation and the acquisition of phoneme categories, in Proceedings of the 27th Annual Boston University Conference on Language Development, Vol. 2, eds Beachley B. Brown A. Conlin F. (Somerville, MA: Cascadilla Press), 650–661.

[B51] Phillips C. Pellathy T. Marantz A. Yellin E. Wexler K. Poeppel D.. (2000). Auditory cortex accesses phonological categories: an MEG mismatch study. J. Cogn. Neurosci. 12, 1038–1055. 10.1162/08989290051137567 11177423

[B52] Polka L. Werker J. F. (1994). Developmental changes in perception of nonnative vowel contrasts. J. Exp. Psychol. Hum. Percept. Perform. 20, 421–435. 10.1037/0096-1523.20.2.421 8189202

[B53] Quilis A. Esgueva M. (1983). Realización de los fonemas vocálicos españoles en posición fonética normal. Estud. Fonética 1, 159–252.

[B54] Saffran J. R. Aslin R. N. Newport E. L. (1996). Statistical learning by 8-month-old infants. Science 274, 1926–1928. 8943209 10.1126/science.274.5294.1926

[B55] Seidl A. Cristia A. (2012). Infants' learning of phonological status. Front. Psychol. 3:448. 10.3389/fpsyg.2012.00448 23130004 PMC3487416

[B56] Seidl A. Onish K. H. Alamian G. Cristia A. (2014). Acoustic correlates of allophonic versus phonemic dimensions in monolingual and bilingual infants' input. J. Phonet. 45, 43–51. 10.1016/j.wocn.2014.03.004

[B57] Shea C. E. (2014). Second language learners and the variable speech signal. Front. Psychol. 5:1338. 10.3389/fpsyg.2014.01338 25477852 PMC4238321

[B58] Shea C. E. Curtin S. (2010). Discovering the relationship between context and allophones in a second language: evidence for distribution-based learning. Stud. Second Lang. Acquis. 32, 581–606. 10.1017/S0272263110000276

[B59] Shea C. E. Curtin S. (2011). Experience, representations and the production of second language allophones. Second Lang. Res. 27, 229–250. 10.1177/0267658310375753

[B60] Shea C. E. Renauld J. (2014). L2 perception of Spanish palatal variants across different tasks. Bilingualism 17, 203–221. 10.1017/S1366728913000047

[B61] Simonet M. Hualde J. I. Nadeu M. (2012). Lenition of /d/ in spontaneous Spanish and Catalan, in Proceedings of Interspeech (Portland, OR: International Speech Communication Association), 1416–1919.

[B62] Stathopoulos E. T. Weismer G. (1983). Closure duration of stop consonants. J. Phonet. 11, 395–400.

[B63] Strange W. Shafer V. L. (2008). Speech perception in second language learners: the re-education of selective attention, in Phonology in Second Language Acquisition, Vol. 36 in Studies in Bilingualism, eds Hansen Edwards J. G. Zampini M. L. (Philadelphia, PA: John Benjamins Publishing Company), 153–191.

[B64] Sussman E. S. Chen S. Sussman-Fort J. Dinces E. (2014). The five myths of MMN: redefining how to use MMN in basic and clinical research. Brain Topogr. 27, 553–564. 10.1007/s10548-013-0326-6 24158725 PMC4000291

[B65] Waltmunson J. (2005). The Relative Difficulty of Spanish/t, d/, Trill and Tap by L1 English Speakers: Auditory and Acoustic Methods of Defining Pronunciation Accuracy. Ph.D. thesis, University of Massachusetts, Amherst, MA.

[B66] Werker J. F. Gilbert J. H. V. Humphrey K. Tees R. C. (1981). Developmental aspects of cross-language speech perception. Child Dev. 5, 349–355. 7238150

[B67] Werker J. F. Tees R. C. (1984). Cross-language speech perception: evidence for perceptual reorganization during the first year of life. Infant Behav. Dev. 7, 49–63.

[B68] Whalen D. H. Best C. T. Irwin J. R. (1997). Lexical effects in the perception and production of American English /p/ allophones. J. Phonet. 25, 501–528. 10.1006/jpho.1997.0058

[B69] White K. S. Peperkamp S. Kirk C. Morgan J. L. (2008). Rapid acquisition of phonological alternations by infants. Cognition 107, 238–265. 10.1016/j.cognition.2007.11.012 18191826 PMC2941201

[B70] Winkler I. Kujala T. Alku P. Näätänen R. (2003). Language context and phonetic change detection. Cogn. Brain Res. 17, 833–844. 10.1016/S0926-6410(03)00205-2 14561466

[B71] Winkler I. Kujala T. Tiitinen H. Sivonen P. Alku P. Lehtokoski A.. (1999). Brain responses reveal the learning of foreign language phonemes. Psychophysiology 36, 638–642. 10442032

[B72] Yeung H. H. Werker J. F. (2009). Learning words' sounds before learning how words sound: 9-month-olds use distinct objects as cues to categorization speech information. Cognition 113, 234–243. 10.1016/j.cognition.2009.08.010 19765698

[B73] Zampini M. L. (1994). The role of native language transfer and task formality in the acquisition of Spanish spirantization. Hispania 77, 470–481.

[B74] Zampini M. L. (1996). Voiced stop spiratization in the ESL speech of native speakers of Spanish. Appl. Psycholinguist. 17, 335–354.

