# Supplementary material for: Establishing New Mappings between Familiar Phones: Neural and Behavioral Evidence for Early Automatic Processing of Nonnative Contrasts
Source: Front Psychol. 2016 Jun 30;7:995. doi: 10.3389/fpsyg.2016.00995 (PMC4928509; doi:10.3389/fpsyg.2016.00995)
Supplement: Supplementary file 1 [file DataSheet1.pdf]

Supplementary Material: Establishing New Mappings between Familiar Phones: Neural and Behavioral Evidence for Early Automatic Processing of Nonnative Contrasts

Shannon L. Barrios [1,*], Anna M. Namyst [2], Ellen F. Lau [2], Naomi H. Feldman [2,3] and William J. Idsardi [2]

[1] *Department of Linguistics, University of Utah, Salt Lake City, Utah, USA*
[2] *Depatment of Linguistics, University of Maryland, College Park, MD, USA*
[3] *Institute for Advanced Computer Studies, University of Maryland, College Park, MD, USA*

Correspondence*:
Shannon L. Barrios
Department of Linguistics, University of Utah, 255 S Central Campus Drive, Rm 2313, Salt Lake City, UT, 84112, USA, s.barrios@utah.edu

Phonology in the bilingual and bidialectal lexicon

1 SUPPLEMENTARY DATA

In order to investigate the possible effect of directionality on the magnitude of the MMN response elicited by each stimulus pair we conducted an additional set of statistical analyses. In our statistical analyses of the listeners' MMN responses to each deviant stimulus, we conducted six planned comparisons separately for each listener group using simultaneous tests for general linear hypotheses with the *multcomp* package in R. P-values were adjusted using the single-step method. Figure 1 shows the mean RMS amplitude of the MMN separated for each contrast by deviant stimulus.

Although these responses are clearly noisier than the summed comparisons due to the reduced power and reversed directionality was associated with some variability in amplitude, the relative pattern of effects across contrasts is largely consistent with the summed comparisons presented in the main document. While some MMN amplitudes were larger in one direction than the other, in no case did we see strong differences in the pattern across contrasts that were dependent on directionality.

As expected for the English listeners, a significant MMN was observed for [iði] deviant when [iɾi] was standard $(\beta = 13.25, SE = 5.17, z - value = 2.56, p = 0.05)$. However, the response to [iɾi] deviants when [iði] was standard, while in the expected direction, did not reach significance $(\beta = 7.96, SE = 5.17, z - value = 1.53, p = 0.51)$. As expected, no MMN was observed for [idi] deviants when [iɾi] was standard $(\beta = 8.81, SE = 5.17, z - value = 1.70, p = 0.39)$, nor was an MMN observed for [iɾi] deviants when [idi] was standard $(\beta = -1.90, SE = 5.17, z - value = -0.36, p = 0.99)$. Unexpectedly, we observed no MMN to [idi] deviants when [iði] was standard $(\beta = 10.09, SE = 5.17, z - value = 1.95, p = 0.25)$, nor to [iði] deviants when [idi] was standard $(\beta = -0.07, SE = 5.17, z - value = -0.01, p = 1.00)$.

For the Spanish group, an unexpected significant MMN was observed for [iði] deviants when [idi] served as standard $(\beta = 11.39, SE = 4.11, z - value = 2.76, p = 0.03)$, but not for [idi] deviants when [iði] was standard $(\beta = 3.83, SE = 4.11, z - value = 0.93, p = 0.91)$. Again, no MMN was observed for either

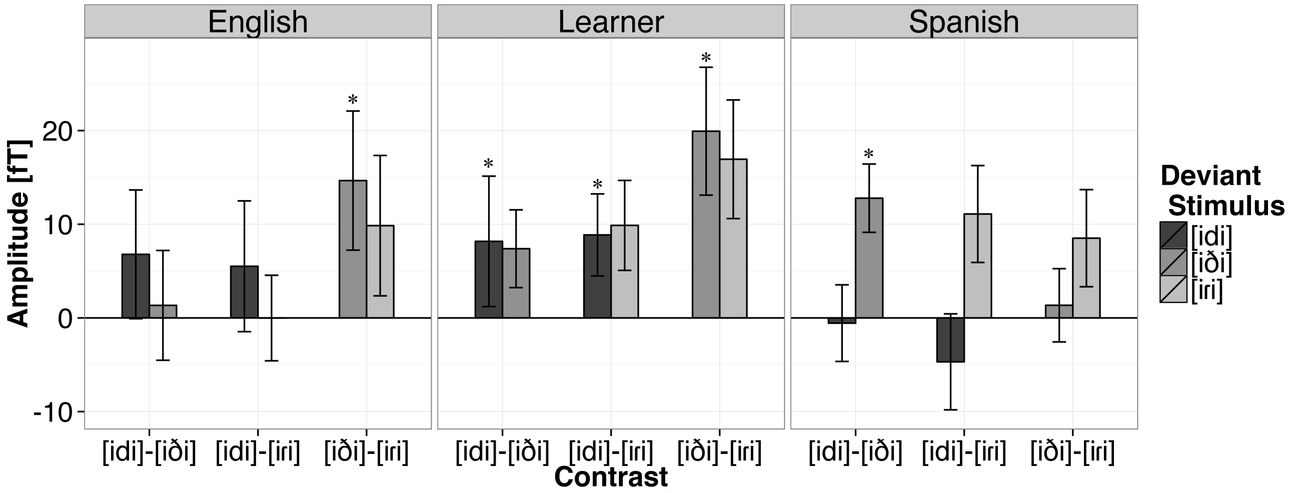

Supplementary Figure 1. Mean RMS amplitude of the MMN [fT] by language group and contrast. Each bar represents the difference between the mean RMS amplitude of the response to a deviant stimulus and the pooled standards when the deviant was presented in a block where the other member of the contrast acts as standard. Error bars represent one standard error of the mean. Asterisks indicate significant MMN responses.

[iði] deviants when [iri] was standard $(\beta = -0.04, SE = 4.11, z - value = -0.01, p = 1.00)$ nor for [iri] deviants when [iði] was standard $(\beta = 5.50, SE = 4.11, z - value = 1.33, p = 0.66)$. No MMN was observed for [idi] deviants among [iri] standards $(\beta = -0.29, SE = 4.11, z - value = -0.07, p = 1.00)$ or vice versa $(\beta = 8.08, SE = 4.11, z - value = 1.96, p = 0.24)$.

For the critical learner group, the MMN results followed the pattern predicted according to the hypothesis that learners successfully implemented the phonological knowledge of their second language at an early, pre-attentive stage of processing. A significant MMN was observed for the L1 allophonic contrast when [idi] deviants are presented among [iði] standards $(\beta = 13.80, SE = 5.17, z - value = 2.66, p < 0.05)$. While no significant MMN was observed for [iði] deviants when [idi] was standard $(\beta = 6.50, SE = 5.17, z - value = 1.25, p = 0.72)$, we again note that the trend is in the expected direction. For the phonemic contrast [idi]-[iri], a significant MMN response is again observed when [idi] deviants are presented in a block where [iri] was standard $(\beta = 14.49, SE = 5.17, z-value = 2.79, p < 0.05)$, but not vice versa $(\beta = 5.12, SE = 5.17, z-value = 0.99, p = 0.88)$. However, again, the observed effect is in the expected direction. A significant MMN response was also observed for Learners' responses to [iði] deviants when [iri] served as standard in the block $(\beta = 14.49, SE = 5.17, z - value = 2.79, p < 0.05)$, but not for [iri] deviants in blocks where [iði] was standard $(\beta = 5.12, SE = 5.17, z - value = 0.99, p = 0.88)$.
